# Mild Benign Paroxysmal Torticollis—A Case Report from Physical Therapy Settings

**DOI:** 10.3390/muscles4020013

**Published:** 2025-05-06

**Authors:** Anna M. Ohman

**Affiliations:** Health and Rehabilitation/Physiotherapy, University of Gothenburg, 405 30 Gothenburg, Sweden; anna.ohman@neuro.gu.se

**Keywords:** benign paroxysmal torticollis, infant, physical therapy

## Abstract

Benign paroxysmal torticollis (BPT) is a condition characterized by episodes of alternating head tilt in infants. Mild cases may be mistaken for Congenital Muscular Torticollis, potentially leading to unnecessary treatment. This case report describes an infant with suspected mild BPT who exhibited alternating head tilt and colic but demonstrated normal motor development. The head tilt resolved spontaneously without intervention. Physical therapists should be aware of mild benign paroxysmal torticollis and monitor such cases carefully to differentiate it from other forms of torticollis and to provide reassurance to parents.

## 1. Introduction

Benign paroxysmal torticollis (BPT) is a condition present in infancy. It is described as periodic episodes of torticollis, a head tilt with head rotation that may alternate sides. BPT was first described by Snyder 1969 [1]. It may be associated with other symptoms, such as vomiting, ataxia, pallor, drowsiness, or irritability [2,3]. Episodes can last from hours to days [3]. The episodes are more frequent and last longer in the earlier months [4]. The condition is self-limiting and resolves spontaneously by 2–3 years of age [2]. In a review from 2024, family history of migraines was found in 25% to 100% of cases [3]. Since 2013, BPT has belonged to a group of “episodic syndromes that may be associated with migraine” [5].

The etiology of BPT is unknown, and the prevalence is difficult to establish as the diagnosis is probably underestimated [3]. Hadjipanayis et al. found in 2015 that child neurologists are aware of BPT, while pediatricians are not. A telephone survey with eighty-two Cypriot pediatricians showed that only 2.4% of them were aware of BPT, and none were confident regarding its management [6]. Bratt and Menelaus conclude that no treatment is indicated for BPT. They recommend that these children are kept under review until the episodes are completely resolved [2]. Neurological findings and motor development have been found to be normal [4,7]. Headaches are common during childhood [8] and BPT has been associated with a small to moderate risk of developing migraines [7,9,10].

In pediatric physical therapy settings, a physical therapist (PT) meets a lot of infants with torticollis, mostly Congenital Muscular Torticollis (CMT). In clinical settings, it is not uncommon to meet infants with torticollis that alters sides; they usually have a normal Passive Range of Motion (PROM) in neck rotation and lateral flexion. Greve et al. found that infants with torticollis that alters sides appear to have a lower cervical range of motion limitations than those that always tilt to the same side [11]. The infants with torticollis that alters sides and possibly without any obvious associated symptoms are likely to have mild BPT.

Unfamiliarity with BTP among pediatricians can delay diagnosis and lead to unnecessary tests and anxiety for parents [6]. Likewise, a PT who is not aware of BTP can give an unnecessary training program, treating BPT as CMT. The PT’s role is primarily to follow progress and motor development, and to react if needed.

There are several differential diagnoses other than CMT and some can be life threatening. Even if these are rare, we need to be aware of them and react early if indicated [12,13,14,15,16].

The Muscle Function Scale (MFS) is a scale constructed to measure muscle function/strength in lateral flexors of the neck in infants with CMT. The MFS uses the head righting response, and scores are given according to the head in relation to the horizontal line. An infant with CMT scores higher on the affected side, the side they tilt toward. [17,18]. Infants that alter sides also show a side difference on the MFS, which the evaluator may interpret as CMT. However, based on the author’s clinical experience, infants with suspected BPT have higher MFS scores on the right side when they tilt toward the right and higher on the left side when they tilt to the left. This means there is probably tension on one side, and this tension alters sides. This may partly be a muscular problem though the underlying mechanism in the muscle during a period of tilting is unknown.

The purpose of describing this case is to share awareness about mild BPT and to avoid unnecessary treatment and cause parental anxiety. It is found that parents of infants with CMT experience stress and anxiety related to the diagnosis and ongoing PT treatment [19]. It is important to follow mild cases of BPT to rule out misdiagnosis.

## 2. Case Description

Initial presentation: An infant aged one and a half months was referred to the clinic for mild plagiocephaly. He had marked hypotonia of the cervical region, a wobbly head that he often held in extension. Parents supported his head all the time, meaning there was no natural training of head control. PROM in neck rotation and lateral flexion was tested and found to be normal. A home training program for head control and tummy time was given.

**Figure 1:**
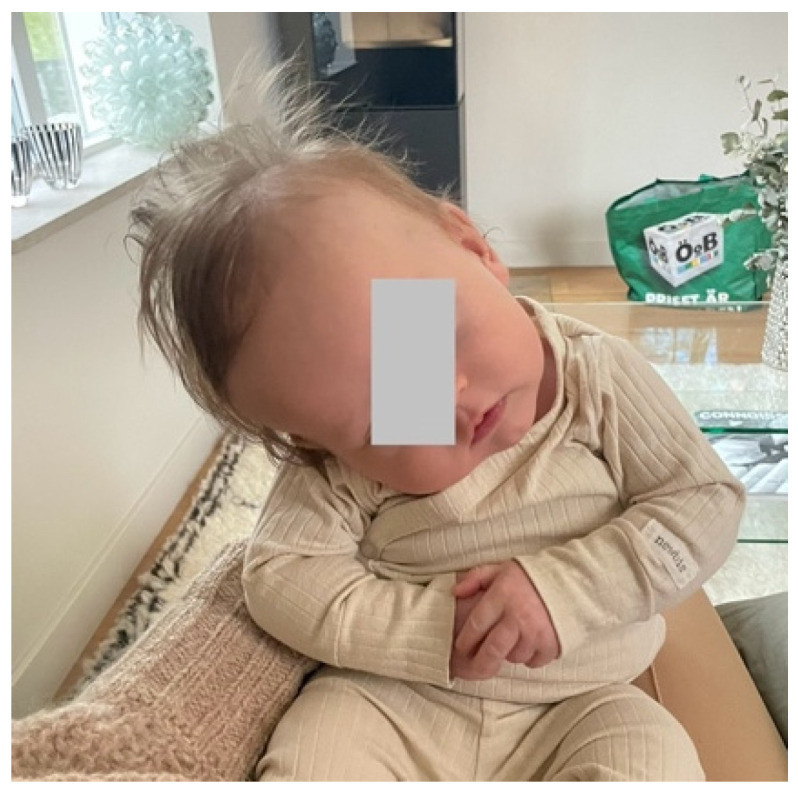
Severe tilting toward his right side.

The infant had colic and vomited a lot according to the parents (his older sister had not vomited at all as an infant); otherwise, he was healthy. At two and a half months, his head control had improved, but at this age he started to tilt his head severely toward the right (Figure 1).

**Figure 2:**
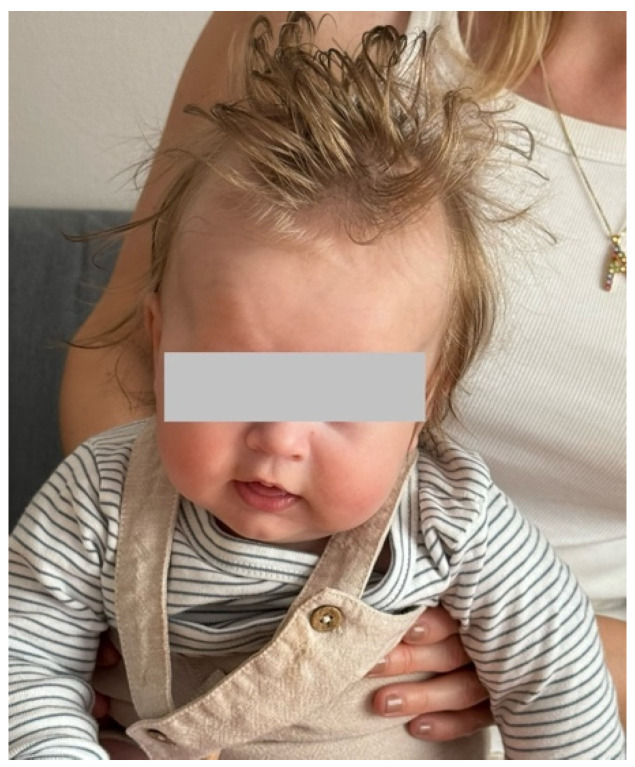
Mild tilting toward his left side.

The tilting toward the right disappeared, and about two weeks later he developed a mild tilt toward the left (Figure 2).

Parents were asked to keep a daily diary of his head tilting, and according to the diary, this alternating pattern continued. Notes about which side the head was tilted and if the tilting was mild or severe were included (Table 1).

Clinical findings: He alternated between the right and left side and between a mild and severe tilt. In the end the tilting was mainly toward the right. The MFS score was always higher on the side he was currently tilting toward. The score changed along with the tilting (Table 2).

The infant’s mother had migraines; there were no other known health problems in the family.

**Figure 3:**
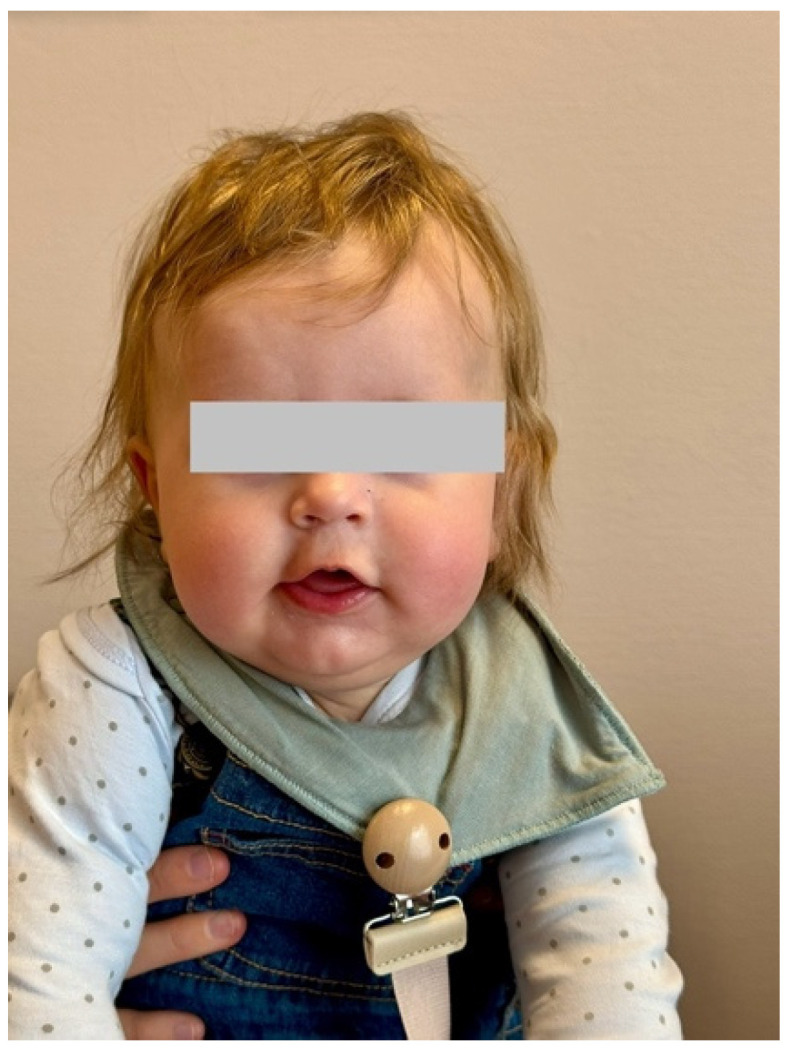
One month before the last visit.

Outcome: At the age of six and a half months, his head was mostly in a straight position (Figure 3).

**Figure 4:**
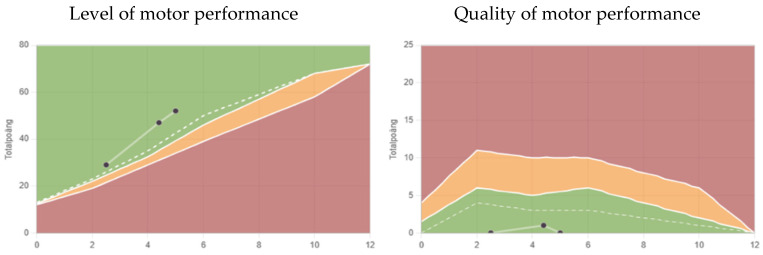
The level of motor performance on the left and quality of motor performance on the right, using the SOMP-I method. Green areas are within the normal span, yellow areas are with a slight delay, and red areas are with a pronounced delay. The white dashed line is the median. The figure shows the case results on three occasions.

Colic had ended at four months of age and the vomiting ended at the same time that he ceased tilting his head. After he gained head control, he had normal motor development (Figure 4); motor function was above the median, calculated using the Structured Observation of Motor Performance in Infants method (SOMP-I). The percentile ranges in SOMP-I are presented as three categories in a “traffic light” system: adequate level of motor performance (green) is on or above the 25th percentile, slight delay (yellow) is between the 6th and 24th percentile, and pronounced delay (red) is on or below the 5th percentile. Adequate quality (green) is on or below the 75 percentile, slight deficit (yellow) between the 76th and 94th percentile and pronounced deficit (red) is on or above the 95th percentile [20,21].

## 3. Discussion

For a pediatric PT, it is not uncommon to meet infants with torticollis that alters sides, possibly without obvious associated symptoms. Some parents say on their first visit that the infant’s head has tilted toward alternate sides. Other parents have not noticed this difference; it is then discovered when they come to the clinic and the infant suddenly tilts to the opposite side compared with earlier records. These infants are suspected to have mild BPT. Bratt and Menelaus described four cases in 1992; the symptoms of the two youngest patients resolved earlier, before one year of age. Only one of them had associated symptoms [2], corresponding well with infants with suspected BPT seen by a PT. Infants with severe attacks are probably referred to a neurologist and not to a PT. It is likely that milder cases, first mistaken for CMT, are referred to a PT.

Today almost all parents have a lot of photos and videos of their infant on their mobile phone. Asking if they want to show these can help evaluate if the infant’s head tilting has altered sides. Asking parents to take new videos or photos of their infant can be a good complement to the written diary of head positions. Rahlin and Sarmiento found still photography reliable when measuring habitual head deviation from midline in infants with CMT [22].

When the MFS shows a side difference in infants with torticollis, we cannot take for granted that it is CMT. Suspected BPT also achieves a higher score on the side the infants currently tilt toward. It is not possible to distinguish between CMT and BPT using only the MFS; however, if the highest score on the MFS alters sides, it could be a sign that it is not CMT. When the highest scores on the MFS alternate, BPT should be considered as one of the possible differential diagnoses. Healthy infants without a head tilt score equal on the right and left side on the MFS [17].

All PTs working with infants, and especially with torticollis, need to be aware of BPT. There is a risk of confusion among professionals in child health care as well as for parents. These infants may receive unnecessary treatment, as recovery is spontaneous. We need to assure parents about the natural progress of BPT and avoid anxiety [4]. However, it would be very interesting to know what happens in the muscle during a period of tilting. As there is a difference on the MFS scale during a period of titling, the muscle is affected in some way. What is the hypothetical explanation for mild BPT? One hypothesis is that the infant may experience comfort when tilting. The question should probably be asked to a pediatric neurologic expert and to a researcher with extraordinary knowledge of morphology.

Minor problems with vomiting, irritability, and colic may be missed if not asked about. Vomiting is not unusual for infants with or without torticollis. Vomiting, irritability, and drowsiness could in some degree be common to any infant. If they only occur when an infant has a tilting episode, they can be regarded as associated symptoms, but if they occur all the time, it may be within the normal variation. If the PROM is normal in the neck, the infant is healthy and no red flags are seen, it is enough to follow the infant monthly. Based on parental anxiety, visits could be sparser in time. In the beginning it may be wise to see the infant more often, until the other reasons for a head tilt are ruled out. The parents must feel reassured and free of anxiety about the diagnosis. To promote motor development, tummy time should be recommended for all infants [22,23,24,25]. At least 30 min of tummy time per day appear beneficial to motor development [26]. This is included in the WHO guidelines for physical activity for infants—30 min spread over the day when the infant is awake [27]. Infant development movement programs are recommended for families and professionals working with infants [25]. In one study, parents reported the recommendation they received for tummy time from nurses at health care centers. The recommendations varied from 0 to >21 min per day [28]. This is not good enough.

### 3.1. Clinical Practice Implications

In a clinic, standardized questions for all infants with torticollis are recommended to catch associated symptoms that may otherwise be missed. However, a careful explanation of what is meant is needed, otherwise there may be a risk of different interpretations.

Suggest questions regard the following topics:

▪ Colic.
▪ Drowsiness.
▪ Irritability.
▪ Vomiting.
▪ Ataxia.
▪ Pallor.
▪ Migraine in the family.
▪ Siblings with any form of torticollis.

Suggested assessment tools are as follows:

- Using a standardized diary to follow head position with the possibility to write comments.
- A photo diary with the infant in a standardized position, e.g., lying in a spontaneous position on the floor, with no attempt from parents to correct the head position, and photos with the infant sitting with support for the torso but no head support.
- Evaluate PROM in neck rotation and lateral flexion.
- Evaluate neck muscles with the MFS.
- Motor development with a proper instrument for infants.

The best way to design a valid and reliable protocol for suspected BPT would be in international cooperation with PTs. Other professions could also be included. Designing and testing a protocol will hopefully give us some insight and an instrument to use in research. It is important to standardize a protocol and make it easy and free to use. Follow up guidelines is also required.

Routine control of motor development for all infants with torticollis could also be included [20,21,29,30] using SOMP-I, a method evaluating motor performance at ages 0–12 months [20,21]. SOMP-I is designed to describe both the level and the quality of motor performance. Another observational examination tool that can be used is the Alberta Infant Motor Scale (AIMS) at ages 0–18 months [29,30]. Both SOMP-I and AIMS track early developmental delays and compare them with the expected norms for the age [20,21,29,30].

### 3.2. Differential Diagnoses

An early exclusion of differential diagnoses is very important as some diagnoses are life threatening [12,13,14,15,16]. The PT must react to red flags presenting in the early stages [31]. For example, torticollis can be the first sign of a posterior fossa tumor in infants [14]. This condition needs early and adequate treatment, and a delay can have severe consequences.

## 4. Conclusions

Mild cases of BPT may be mistaken for CMT and referred to a PT. The PT needs to distinguish BPT from CMT at an early stage to avoid unnecessary treatment. It is important to follow infants with BPT to exclude more severe causes of torticollis. An infant that does not seem to be healthy, e.g., is sensitive to normal light or is developing irregularly, should be referred to a physician. Further studies are needed to obtain better knowledge of morphological changes in the sternocleidomastoid muscle in infants with suspected BPT. Designing a standardized protocol to evaluate suspected BPT is of great importance.

## Figures and Tables

**Table 1:** Diary of head positions—left, right or straight. Degree of tilt recorded only in terms of mild or severe to make it easier for the parents to judge.

Day Starting Diary	Tilt of Head Toward Left or Right and Mild or Severe
1	Left severe
2	Right mild
3	Right mild
4	Right mild
5	Right mild in morning, afternoon left mild
6	Right mild in morning, afternoon left mild
7	Left severe
8	Left mild morning, rest of the day right mild
9	-
10	Both left and right, afternoon and morning
11	-
12	Both left and right, afternoon and morning
13	Right severe
14	Right mild
15	Left mild
16	Straight
17	Right mild
18	Right severe
19	Right mild
20	Right severe
21	Right mild
22	Right mild
23	Left severe
24	Right mild
25	Right mild
26	Right severe
27	Right mild
28	First right then changed to left
29	Right mild
30	Right mild
31–46	Three days to the left and 12 days to the right alternating from mild to severe

**Table 2:** MFS scores taken on six of the visits, always a higher score on the side that the head was currently tilting toward. When straight no difference in scores.

Head Position	MFS Score Right	MFS Score Left
Tilt right	3	1
Tilt right	4	3
Tilt left	2	3
Tilt right	4	2
Tilt left	3	5
Head straight	4	4

## Data Availability

The data presented in this study are available on request from the author.
